# Characteristics of Exposure of Reproductive-Age Farmworkers in Chiang Mai Province, Thailand, to Organophosphate and Neonicotinoid Insecticides: A Pilot Study

**DOI:** 10.3390/ijerph17217871

**Published:** 2020-10-27

**Authors:** Neeranuch Suwannarin, Tippawan Prapamontol, Tomohiko Isobe, Yukiko Nishihama, Shoji F. Nakayama

**Affiliations:** 1 Research Institute for Health Sciences (RIHES), Chiang Mai University, Chiang Mai 50200, Thailand; suwannarin.ns@gmail.com; 2 Centre for Health and Environmental Risk Research, National Institute for Environmental Studies, Tsukuba, Ibaraki 305-8506, Japan; isobe.tomohiko@nies.go.jp (T.I.); nishihama.yukiko@nies.go.jp (Y.N.)

**Keywords:** organophosphate, dialkylphosphate, neonicotinoid, insecticide, metabolite, occupational exposure, human biomonitoring, urine, reproductive age, farmworker

## Abstract

Exposure to insecticides containing organophosphate (OP) and neonicotinoid (NEO) compounds has been associated with adverse reproductive health outcomes. This study characterized and identified predictors of exposure to OP and NEO among 100 reproductive-age farmworkers from two intensive farming areas in Chiang Mai Province, Thailand, including 50 each from the Fang (FA) and Chom Thong (CT) districts. OP exposure was determined by measuring the urinary concentrations of six dialkylphosphates (DAPs), whereas NEO exposure was determined by measuring the urinary concentrations of NEO compounds and their metabolites (NEO/m). The most frequently detected OPs were diethylphosphate (DEP) and diethylthiophosphate (DETP), with DETP having the highest geometric mean (GM) concentration, 8.9 μg/g-creatinine. The most frequently detected NEO/m were N-desmethyl-acetamiprid (N-dm-ACE), imidacloprid (IMI), and thiamethoxam (THX), with IMI having the highest GM concentration, 8.7 μg/g-creatinine. Consumption of well water was the predominant determinant of OP and NEO exposure in this population. In addition to encouraging workers to use personal protective equipment, exposure of farmworkers to these compounds may be reduced by nation-wide monitoring agricultural insecticides and other pesticides in community drinking water resources.

## 1. Introduction

Agricultural practices in Thailand have changed from traditional to agrochemical-based commercial cultivation, with a 1.8-fold increase in imported pesticides over the past decade, from 109,908 tons in 2008 to 198,317 tons in 2017, resulting in increased productivity [1]. Approximately 32% of the total workforce is reported as engaged in agriculture, while 47% percent of the total land area is used for agricultural production in Thailand [1,2]. Thailand has long been a major exporter of commodities and in 2016, the exports of rice and natural rubber accounted for 28% of the total value of agricultural exports, followed by fruits and fruit products, accounting for 11%. Thai farmers rarely use personal protective equipment, leading to increasing risk of pesticide exposure [3]. Organophosphate (OP) insecticides are ubiquitously used in agriculture and in residential applications, with chlorpyrifos being the leading imported insecticide in Thailand [2]. Recent studies conducted in Thailand have measured the urinary dialkyl phosphates (DAP), common metabolites of OP, in many regions, and the results may vary depending on characteristics of study areas and crop cultivation. For instance, vegetable farmers in the northeastern region had urinary DAP concentration higher than vegetable farmers in the northern region [4,5]. Moreover, the concentration of urinary DAP detected in Northern Thai farmers engaged in different types of crop cultivation have a different concentration from low to high exposure [5,6]. Neonicotinoid (NEO) insecticides have been replacing conventional OP, carbamate, and pyrethroid insecticides, as they have wide spectrum activity, low volatility, and highly selective insecticidal properties, as well as being less toxic to mammals [7,8]. Worldwide, approximately 60% of NEO insecticides are applied to seeds and soil in the cultivation of several crops, including corn, cotton, citrus fruits, rice, and soybeans [7]. The use of OP and NEO insecticides has continually increased. Although OP exposure has been widely studied [6,9,10,11,12], less is known about the NEO exposure.

OP exposure has been monitored in human subjects, as have the effects of long-term exposure to OP [13,14,15]. Exposure of adults to OP insecticides has been linked to poor semen quality, infertility, and other reproductive problems [16,17,18]. Moreover, prenatal exposure to OPs has been linked to adverse outcomes at birth and on children’s health, including reduced birth weight and head circumference [19,20,21], attention-deficit hyperactivity disorder [22], and neurodevelopmental abnormalities [23].

Maternal exposure to NEO has also been associated with adverse outcomes, including autism spectrum disorder and anencephaly [24,25,26]. NEO can be transferred from mothers to fetuses, resulting in low birth weight infants [27]. These findings have increased concerns over the association between exposure to NEOs and adverse health effects, particularly among young adults. Several studies have assessed NEO exposure and concentrations of their metabolites in different target populations. For example, Japanese children and adults, as well as populations living in different regions of China, can be exposed daily to NEO [28,29,30]. To our knowledge, however, urinary concentrations of NEO have not been measured in populations living in Thailand. This pilot study therefore attempted to characterize and identify the predictors of exposure to OP and NEO insecticides in reproductive-age farms workers living in two intensive agricultural areas of Chiang Mai Province, Thailand.

## 2. Materials and Methods

### 2.1. Study Participants and Sample Collection

**Figure 1:**
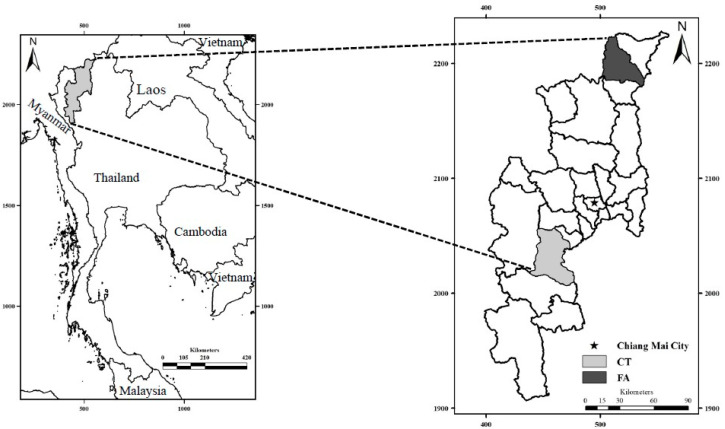
Maps of Thailand (left) and Chiang Mai Province, showing the Fang (FA) and Chom Thong (CT) districts, located at 19°54′26″ N, 99°2′26″ E and 18°25′2″ N, 98° 40′27″ E, respectively.

This cross-sectional study assessed reproductive-age farmworkers working in Fang (FA) and Chom Thong (CT) Districts of Chiang Mai Province, Thailand, in February 2018. The FA and CT Districts are located about 154 km north and 70 km south of Chiang Mai City, respectively, and are both areas of intensive agriculture (Figure 1).

This study included 50 farmworker couples, 25 from each district, of reproductive age (aged 18 to 40 years), who worked at least 6 months in the study areas. Women were included only if they were not pregnant at recruitment, as confirmed by pregnancy test strip. All participants signed informed consent forms prior to urine sampling and data collection. Each subject provided spot urine samples of about 50 mL, each of which was aliquoted into 10-mL and 5-mL polypropylene tubes, cooled on ice and transferred to the Toxicology Laboratory, Research Institute for Health Sciences (RIHES), Chiang Mai University and stored at −20 °C until analyzed. In addition, 5 mL of each urine sample were shipped on dry ice to the Center for Health and Environmental Risk Research, National Institute for Environmental Studies (NIES), Tsukuba, Japan and stored at −80 °C until analyzed.

The participants were interviewed face-to-face using a structured questionnaire. Factors recorded included sociodemographic factors (i.e., age, gender, body mass index [BMI], family income, educational level, marital status, and parity) and factors associated with work and exposure to pesticides (i.e., use of residential pesticides, years working as a farmworker, time working in the field, occupational status, farm tasks, farm tasks related to pesticide use, and use of personal protective equipment [PPE]) Also included were questions about food consumption, including fruits and vegetables and the main sources of water. The study protocol was approved by the ethics committee of the Research Institute for Health Sciences, Chiang Mai University (Project no. 14/60, approved on 27 October 2017) and by the Institutional Review Board of the National Institute for Environmental Studies, Japan (NIES2018-003, approved on 23 July 2018).

### 2.2. Chemicals and Reagents

#### 2.2.1. Analyses of Urinary OP Metabolites

Analytical grade methanol, acetone, ethyl acetate, acetonitrile, hexane, and toluene were all purchased from J.T. Baker (Phillipsburg, NJ, USA). Concentrated hydrochloric acid (HCl, 36–38%, 6 N) was purchased from Merck (Darmstadt, Germany). Sodium chloride (NaCl, purity ≥ 99.5%), potassium carbonate (K2CO3, purity ≥ 99.0%) and 2,3,4,5,6-pentafluorobenzyl bromide (PFBBr, purity 99.0%) were purchased from Sigma Aldrich (St. Louis, MO, USA). Dimethylphosphate (DMP, purity 97.0%), sodium *O,O*-dimethyl thiophosphate (DMTP, purity 95.0%), *O,O*-dimethyl dithiophosphate (DMDTP, purity 95.0%), *O,O*-diethyl hydrogen phosphate (DEP, purity 96.0%), *O,O*-diethyl dithiophosphate (DEDTP, purity 95.0%), and *O,O*-diethylthiophosphate (DETP) potassium salt (purity 98.0%) were purchased from Chiron AS (Trondheim, Norway). Dibutyl phosphate (DBP, purity 99.0%), used as the internal standard, was purchased from Toronto Research Chemicals (Toronto, ON, Canada). Type I ultrapure water was generated in-house using an ELGA UHQPSII system (Buckinghamshire, UK).

#### 2.2.2. Analyses of Urinary NEO and Their Metabolites (NEO/m)

Standard stock solutions of unlabeled native neonicotinoid mixtures and internal standards were purchased from Cambridge Isotope Laboratories (Tewksbury, MA, USA). Methanol (purity 99.8%) was purchased from Nacalai Tesque, Inc. (Kyoto, Japan). Acetonitrile (purity 99.8%), ammonium acetate (purity 97.0%), and formic acid (purity 99.0%) were purchased from Wako Pure Chemical Cooperation (Osaka, Japan). ISOLUTE^®^ HYDRO DME + 400 mg Plates were purchased from Biotage (Uppsala, Sweden). Milli-Q ultrapure water was prepared using a Millipore Milli-Q INTEGRAL 5 A10 Water Purification System (Merck, Darmstadt, Germany).

### 2.3. Analyses of Urinary Metabolites

#### 2.3.1. Analyses of Urinary OP Metabolites

Concentrations of urinary DAP metabolites including dimethylphosphate (DMP) and dimethylthiophosphate (DMTP), diethylphosphate (DEP), diethylthiophosphate (DETP), and diethyldithiophosphate (DEDTP) were measured by gas chromatography with a flame photometric detector (GC-FPD) [31]. Brief, a 50-µL aliquot of 1.25 mg/L DBP, utilized as an internal standard, was added to a 5-mL urine sample in a screw-top glass test tube containing 2 g NaCl. To each sample was added 1 mL 6 M HCl, followed by vigorous extraction with 5 mL of ethyl acetate: acetone (1:1, *v*/*v*). After shaking, the samples were centrifuged for 3 min at 2000× *g* and extracted with 5 mL of ethyl acetate. The supernatants were combined in a round bottle containing 20 mg K_2_CO_3_, followed by evaporation at 37 °C. Each dried residue was re-dissolved three times with 3 mL acetonitrile each in a screw-top glass tube containing 20 mg K_2_CO_3_. After derivatization with PFBBr at 50 °C for 15 h, the derivatized target metabolites were extracted with 4 mL type I ultrapure water and twice with 5 mL hexane. After shaking, the samples were centrifuged for 3 min, and the solutions were combined. Each extract was evaporated under a gentle stream of nitrogen and re-dissolved in 50 µL toluene. These solutions were subsequently analyzed by gas chromatography with a flame photometric detector (Hewlett Packard 6890-FPD, Agilent Technologies, Santa Clara, CA, USA).

#### 2.3.2. Analyses of Urinary NEO/m

Urinary NEO/m was analyzed at NIES by liquid chromatography-tandem mass spectrometry (LC-MS/MS). Briefly, 10 µL of internal standard solution were added to 100 µL of each urine sample. To each was added 600 µL of acetonitrile to precipitate proteins, followed by centrifugation to obtain the supernatants. These supernatants were loaded onto plates preconditioned with 100 µL acetonitrile, followed by centrifugation at 2000× *g* for 1 min. The samples were evaporated to dryness with a gentle N2 stream at 45 °C for 15 min. Each residue was dissolved in 200 µL 0.1% formic acid containing 10 mM ammonium acetate and methanol (95:5, *v*/*v*), and the samples were mixed for 30 s. Each eluate was loaded onto a Nexera liquid chromatograph system coupled to a Triple Quad 8060 mass spectrometer (Shimadzu Corporations, Kyoto, Japan).

### 2.4. Quality Assurance and Quality Control

#### 2.4.1. Analyses of Urinary OP Metabolites

Pooled urine samples made from anonymous non-farming volunteers were fortified with a standard solution containing DMP (75 ng/mL), DEP (20 ng/mL), DMTP (8 ng/mL), DMDTP (4 ng/mL), DETP (4 ng/mL), and DEDTP (8 ng/mL). The resulting quality control (QC) samples were analyzed for precision of reproducibility. Five replicates of QC samples were analyzed by a single operator in a single day for within-day precision. For between-day precision, five QC samples were analyzed by a single operator on three consecutive days. The recoveries of QC samples ranged from 92.7% to 112%, with relative standard deviations (RSDs) for within-day precision ranging from 9.1% for DMDTP to 15.2% for DMP, and RSDs for between-day precision ranging from 7.9% for DETP to 13.0% for DMP. Five calibration points were set to range from 25–125 ng/mL for DMP, from 5.0–40 ng/mL for DMDTP and DEP, from 2.0–32 µg/mL for DMTP and DEDTP and from 1.0–16 ng/mL for DETP, whereas the internal standard concentration was 25 µg/mL. All sample measurements were performed in triplicate to determine the reliability of the measurement. Each sample was prepared three times and analyzed, and the resulting values were averaged. Proficiency testing materials from the German External Quality Assessment Scheme (G-EQUAS) were analyzed as part of quality assurance of the method (Table S1). Overall, the reported values fell well within the tolerance ranges.

#### 2.4.2. Analyses of Urinary NEO/m

Urine samples were collected from pregnant volunteers from Japan and pooled as a QC sample. The QC sample was spiked with known concentrations of NEO/m standard. These samples were analyzed as part of quality quarantine and recorded in a Shewhart control chart (X-Rm control chart) based on ISO 7870. The method detection limit (MDL) was estimated from the equation:

MDL = *t*_(*n* − 1, 0.05)_ × 2 × *s* (1)

where *t*_(*n* − 1, 0.05)_ represents Student’s t value at an α level of 0.05 with *n* − 1 degrees of freedom, and s represents the standard deviation (SD) of blank measurements in *n* replicates (*n* ≥ 7).

### 2.5. Statistical Analysis

Subject-related sociodemographic factors and work and exposure characteristics of the study population were reported as frequency distribution or mean ± standard deviation (SD). Urinary concentrations of individual DAP metabolites and NEO/m, expressed as μg/L, were normalized relative to creatinine concentrations in the same samples to adjust for urine dilution; these concentrations were therefore reported as μg/g-creatinine. DAP and NEO/m concentrations were log10-transformed to obtain normal distributions before statistical analyses. Summary statistics were computed using the NADA package (version 1.6-1.1) in the statistical R software. Geometric means (GM) and geometric standard deviation (GSD) were calculated for analytes detected in >50% of samples. Selected percentiles, including 50%, 75%, 95%, and maxima, were also determined.

Urinary concentrations below the MDL were determined by the multivariate imputation by chained equations (MICE) method, with 10 imputations and 10 iterations prior to multivariate linear regression analysis. Multivariate linear regression analyses were performed to select predictors of urinary DEP, DETP, imidacloprid (IMI), and N-desmethyl-acetamiprid (N-dm-ACE) concentrations using a backward stepwise multiple linear regression, setting an alpha level at 0.05. The following independent variables were finally selected: sociodemographic characteristics of the study population, including age, gender, BMI, study site area, family income, and educational level; occupational exposure, including use of residential pesticides, occupational status, farm tasks, farm tasks related to pesticide use and PPEs used; and food consumption, including vegetables and fruits consumed, and main source of water for consumption. The regression coefficient (β) of multivariable models represented the average increase of each dependent variable in response to a one unit increase of each independent variable. All statistical analyses were performed using the statistical R software version 4.0.1 [32].

## 3. Results

### 3.1. Sociodemographic Characteristics of the Study Participants

Table S2 summarizes the sociodemographic characteristics of the 100 farmworkers of reproductive age participating in this study. Their mean ± SD age was 30.1 ± 5.6 years and their mean ± SD BMI was 24.4 ± 3.9 kg/m^2^. An estimated 34% of the participants were of normal weight, 19% were overweight, 31% were of obese class I, 10% were of obese class II, and 6% were underweight based on the Regional Office for the Western Pacific (WPRO) standard [33]. Of the participants, 31% had completed high school, 29% had no formal education, 26% had completed primary school, and 14% had more than a high school education. About 60% of farmworkers self-identified as Northern Thai and 40% as other ethnicities. The mean monthly family income was about 320 USD (about 10,012 THB).

Table S3 summarizes the work and exposure characteristics of these farmworkers. On average, these farmworkers had engaged in agriculture for about 12.3 years, typically working 6.0 days per week for about 8.4 h per day. More than 70% worked on their own farms, 10% worked on farms that they rented, and 17% worked on others’ farms. Over 70% used residential pesticides. The main crops produced on farms in the FA district were citrus fruits, corn, and lychee, whereas the main crops produced on farms in the CT district were ornamental cut flowers, particularly chrysanthemums (data not shown). The agricultural activities of these farmworkers are shown in Table S4 and their PPE in Table S5. Of these individuals, 86% wore long-sleeved shirts/pants, 83% wore boots, 78% wore masks, 66% wore gloves, 53% wore hats or scarves as head protection, and 9% wore eyeglasses.

### 3.2. Urinary DAP and NEO/m Concentrations

The urinary concentrations of OP and NEO/m are shown in Table 1. Of the six OP metabolites assayed, DEP and DETP were the most frequently detected, being present in 100% of urine samples. Moreover, DETP had the highest GM concentration (8.9 μg/g-creatinine) and the highest maximum GM concentration (261 μg/g-creatinine). In comparison, the GM concentrations of DEP and DEDTP were 6.7 and 7.3 μg/g-creatinine, respectively.

N-dm-ACE, IMI, and thiamethoxam (THX) were detected in 99%, 94%, and 69% of urine samples, respectively, followed by CLO in 59% and Of-IMI, a metabolite of IMI, in 50%. The other NEO/m compounds were detected in ≤ 34% of urine samples. IMI had the highest GM concentration, 8.7 μg/g-creatinine, followed by N-dm-ACE (7.3 μg/g-creatinine), THX (4.3 μg/g-creatinine), imidacloprid-olefin (Of-IMI) (2.6 μg/g-creatinine) and clothianidin (CLO) (2.4 μg/g-creatinine). Of-IMI had the highest maximum detected urinary concentration (296 μg/g-creatinine).

### 3.3. Potential Predictors of Exposure

Table 2 presents the potential predictors of DEP, DETP, IMI, and N-dm-ACE concentrations. Farm tasks, farm task-related pesticide used, and PPE used were excluded from the final multivariable model as their adjusted *p*-values exceeded 0.1. The final multivariate model for predictors of DAP and NEO/m exposure was adjusted for gender, age, BMI, study site, educational levels, family income, occupational status, use of residential pesticides, vegetables and fruits consumed, and source of water consumption. Increased age tended to be associated with increasing DEP concentrations (*p* = 0.053), and urinary concentrations of N-dm-ACE were significantly lower in men than in women. Farmworkers in the Fang district had significantly higher DEP (*p* = 0.003) and marginally higher DETP (*p* = 0.054) concentrations than workers in the Chom Thong district. Educational levels were significantly positively associated with N-dm-ACE concentrations. IMI concentration were marginally higher in those who worked on others’ farms than in those who worked on their own farms (*p* = 0.053). Well water as the main source of drinking water was associated with increased concentrations of DEP, DETP, and IMI, and vegetable consumption tended to be associated with higher DEP concentrations (*p* = 0.050). Overall, the consumption of well water was identified as the strongest predictor of DAP and NEO/m concentrations in multivariate models.

## 4. Discussion

This study assessed exposure to OP and NEO insecticides of reproductive-age farmworkers from two districts in Chiang Mai Province, Thailand, by measuring urinary concentrations of DAP and NEO/m. We found that these individuals were ubiquitously exposed to OP and NEO insecticides. The most frequently detected OP metabolites were DEP, DETP, and DEDTP, whereas the most frequently detected NEO/m compounds were N-dm-ACE, IMI, and THX. We found that drinking well water was the main contributor to urinary DAP and NEO/m concentrations. In addition, sex, age, study site, educational level, occupational status, and vegetable consumption were associated with urinary DAP and NEO/m concentrations.

### 4.1. OP

#### 4.1.1. Urinary OP Metabolites

The present study found that the diethyl moiety was the most frequently detected (100%), with the methyl moiety detected in ≤36% of samples. These findings differed from those of studies in Japan and the United States, which was likely due to the OP insecticide used in Japan and United States consisting primarily of compounds with the dimethyl moiety such as fenthion and methamidophos [34,35]. The main OP pesticides used in the FA district, including chlorpyrifos, ethion, and profenofos, are primarily metabolized to diethyl compounds [20,36]. The OP metabolite with the highest urinary concentration in the current study was DETP (8.9 μg/g-creatinine). This concentration was much higher than that previously reported in Thai farmers (0.86 μg/g-creatinine), a difference probably due to the intensive use of insecticides in our study areas [37]. Although higher concentrations of DAP metabolites reflect greater exposure to parent OP insecticides, the metabolites cannot be directly sourced to specific OP insecticides [38]. Further studies that include measurements of parent OP pesticides are needed to identify possible sources of OP metabolites in biological samples.

#### 4.1.2. Potential Predictors of Urinary OP Metabolites

The major predictor of high urinary concentrations of diethyl group-containing OP metabolites in this population of reproductive-age farmworkers was consumption of local well water. This finding is consistent with the results of a birth cohort study in the FA district, which found that source of water consumption was associated with increased concentrations of diethyl group containing compounds [20]. Urinary DEP and DETP concentrations were higher in the 6% of farmworkers who drank well water than in subjects who did not, suggesting that this well water was contaminated by diethyl OP insecticides. Although this finding was consistent with a study from California [39], a study of schoolchildren in Chile found that drinking water was not a source of OP exposure, as no OP insecticide residues were detected in the water samples taken from these children’s schools and homes [40]. Although the higher DEP and DETP concentrations in the FA district may have been due to well water intake by these farmworkers, we did not collect well water samples in the two study areas or measure their concentrations of contaminants. Additional studies are needed to quantify OP pesticides in water for consumption.

The finding, that urinary DETP concentrations were marginally higher in farmworkers who did than did not consume vegetables, was consistent with previous results [41]. Although vegetable consumption may be a source of farmworkers’ exposure to pesticides, our findings were based on the results of face-to-face questionnaires. Better approaches are needed to assess fruit and vegetable consumption, such as a validated food frequency questionnaire. Other factors found to be potentially predictive of OP exposure include smoking status, fasting at the time of urine sampling, season of the year, living in proximity to agricultural farms and consumption of specific dietary items [40,42,43,44]. However, the present study did not assess these factors.

### 4.2. NEO

#### 4.2.1. Urinary NEO/m Concentrations

To our knowledge, the present study is the first to report the urinary concentrations and distributions of NEO/m in Thailand, particularly in reproductive-age farmworkers. The most frequently detected NEO/m was IMI, which also had the highest GM concentration (6.3 μg/g-creatinine). This concentration was higher than in populations from the US (0.074 μg/g-creatinine) [45] and Japan (1.54 μg/g-creatinine) [28], which may have been due to the occupational exposure of Thai farmworkers to NEO/m in the present study. In contrast, the concentration of IMI was much higher in a population from Henan, China (10.5 μg/g-creatinine) than in our study, a difference that may have been due to greater NEO application in China, consisting of three to four IMI spraying events [46]. Reports by the Thailand pesticide alert network (Thai-PAN) about imported pesticides indicate that IMI is the largest proportion among NEO groups [47]. Moreover, the farmworkers in our study may have been exposed to NEO/m through consumption of foods such as fruits and vegetables [48]. We found that these farmworkers were frequently exposed to several NEO, especially IMI, ACE, THX, ACE, and CLO, consistent with results reported by the Thai-PAN. Indeed, several NEO compounds, including IMI, ACE, THX, CLO, and dinotefuran (DIN), were detected in honey oranges collected in 2014–2018 [47]. The continuous, widespread use of NEO over the last decades was consistent with the compounds detected in tangerines collected from the Chiang Mai Tangerine Cluster in 2007 [49]. Because NEO exposure has not been assessed in other regions of Thailand, additional studies are needed to evaluate nationwide exposure.

The finding that male sex was associated with lower urinary N-dm-ACE concentrations is consistent with results showing that the 95th percentile of N-dm-ACE concentrations was lower in men (1.24 μg/g-creatinine) than in women (2.22 μg/g-creatinine). Moreover, the ratio of N-dm-ACE concentration to that of its parent ACE was higher in women than in men, suggesting that this difference may be associated with gender-related differences in metabolism [50]. Lower concentrations in men may also be associated with the higher rates of insufficient fruit and vegetable intake in Thai men than women [51]. Further studies, however, are needed to determine the reason for these gender-related differences.

#### 4.2.2. Potential Predictors of Urinary NEO/m Concentrations

The higher urinary IMI concentrations in farmworkers who consumed well water suggest that this well water was contaminated with NEO insecticides, a finding consistent with the results of studies in Canada and China [52,53]. Drinking water can be contaminated by the migration of NEO from agricultural lands into water sources. NEO exposure in our population may have been due to their consumption of contaminated well water. This finding was consistent with the results of a Japanese study, in which adults were found to be exposed to NEO primarily by the drinking water in their daily lives [30,54]. Additionally, the study in China reported that children who consumed tap water and fresh vegetables were more likely to have urinary total NEO [55]. To our knowledge, no studies have evaluated NEO/m residues in drinking water, fruits, and vegetables in Thailand, suggesting the need for future studies to include sampling of water and foods in our study areas. Dietary items, such as specific fruits and vegetables, and physical exercise behavior were found to be potential predictors of NEO exposure [55], but these factors were not determined in our study.

### 4.3. Limitations

This pilot study had several limitations. First, exposure to pesticides was determined by taking a single spot urine sample from each participant. Because OPs have a short biological half-life, urinary concentrations of DAP reflect recent exposure, i.e., within 6–24 h [56]. Because DAPs are non-specific metabolites of OP, we could not evaluate exposure to specific OPs. In addition, DAPs can be found in foods [44], indicating that we may have overestimated exposure from urinary DAP concentrations. However, spot urine sample collected from children on three different days revealed substantial reliability, with intraclass correlation coefficients (ICC) greater than 0.8, indicating that spot urine was adequate for estimating exposure to OP [57]. Although NEOs also have a short half-life [58], we included the major metabolites of their parent compounds, which have been recommended as biomarkers for human exposure to NEO [47]. Spot urine samples from subjects in the US showed poor reliability with ICCs for concentrations of NEOs, including IMI, THX, CLO, and N-dm-ACE, ranging from 0.09 to 0.42, suggesting that single spot urine samples may not adequately represent exposure for over a month [59]. Another limitation of this study was the small sample size. Larger scaled studies should include more detailed information about food consumption data and participants’ behavior.

From these findings, farmworkers may be exposed to pesticides, including both OP and NEO, through multiple routes, not only spray drift but ingestion through eating or drinking. The current data regarding the source of water intakes should be made available to their communities, to raise awareness and to avoid intake of untreated water, or water runoff directly from agricultural farms to their villages. The data from the present study can be applied for further studies in similar agricultural areas to investigate exposure as well as its plausible predictor.

## 5. Conclusions

The present study revealed that Thai reproductive-age farmworkers living in intensive agricultural areas of Chiang Mai Province are exposed to both OP and NEO. The high detection frequencies were DEP and DETP with the highest GM concentration being DETP (8.9 μg/g-creatinine), indicating the high use of diethyl moiety OP insecticides. N-dm-ACE, IMI, and THX are the dominant NEO/m, with the highest GM concentration obtained for IMI (8.7 μg/g-creatinine). This is the first to report on characteristics of exposure to NEO insecticides in Thai adult farmworkers. This study also provides valuable information on the major determinants of occupational OP and NEO exposure in farmworkers. Drinking well water was found to be the major predictor of urinary diethyl DAP, including DEP, DETP, and IMI, in farmworkers. Sociodemographic data and food consumption were found to be predictive of exposure to OP and NEO/m. Since this is a pilot study before designing the larger scaled study, we did not collect sufficient information. Our study is also limited due to its small sample size, thus the results are still inconclusive. Further studies using a larger sample size are warranted to elucidate the predictor that may play a possible role as the source of exposure and concern about its reproductive health effects. These results also suggest the importance of PPE use in reducing pesticide exposure, as well as nationwide monitoring of the contamination of community drinking water resources with agricultural insecticides and other pesticides.

## Figures and Tables

**Table 1:** Urinary DAP and NEO/m concentrations in reproductive-age farmworkers (*N* = 100).

Compounds	Reproductive-Age Farmworkers (*N* = 100)							
(μg/g Cr)	%> MDL	GM	GSD	95%CI	P50	P75	P95	Max.
DAP:								
DMP	15.0	-	-	-	-	-	17.3	91.2
DMTP	36.0	-	-	-	-	1.5	9.3	239.3
DMDTP	11.0	-	-	-	-	-	1.5	102.1
DEP	100.0	6.7	2.4	5.7–8.0	4.3	7.6	20.4	126.1
DETP	100.0	8.9	3.2	7.1–11.3	3.7	10.2	30.5	261.3
DEDTP	65.0	7.3	5.7	5.2–10.2	1.1	2.1	9.4	125.3
NEO/m:								
ACE	34.0	-	-	-	-	0.008	0.05	0.3
CLO	59.0	2.4	2.5	2.0–2.8	0.06	0.1	0.4	1.2
DIN	9.0	-	-	-	-	-	0.06	0.3
IMI	94.0	8.7	4.2	6.6–11.6	0.3	0.7	2.7	75.3
NIT	0.0	-	-	-	-	-	-	-
THX	69.0	4.3	4.2	3.2–5.7	0.04	0.1	1.3	6.6
THI	0.0	-	-	-	-	-	-	-
FLN	2.0	-	-	-	-	-	-	0.2
N-dm-ACE	99.0	7.3	2.6	6.0–8.8	0.3	0.7	2.6	5.6
N-dm-THX	0.0	-	-	-	-	-	-	-
OH-IMI	0.0	-	-	-	-	-	-	-
Of-IMI	50.0	2.6	3.5	2.0–3.3	1.2	3.4	13.3	296.6
SUF	2.0	-	-	-	-	-	-	1.0
THIAM	3.0	-	-	-	-	-	-	0.01
TFNA-AM	0.0	-	-	-	-	-	-	-
TZNG	5.0	-	-	-	-	-	-	0.5
Fipronil	0.0	-	-	-	-	-	-	-
Fipronil sulphide	0.0	-	-	-	-	-	-	-
Fipronil sulphone	8.0	-	-	-	-	-	0.02	0.05
Ethiprole	0.0	-	-	-	-	-	-	-

Abbreviations: MDL, method detection limit; μg/g, micrograms per gram; Cr, creatinine; GM, geometric mean; GSD, geometric standard deviation; CI, confidence Interval; P50, 50th percentile; P75, 75th percentile; P95, 95th percentile; Max., maximum concentration; DAP, dialkylphosphate; DMP, dimethylphosphate; DMTP, dimetylthiophosphate; DMTDP, dimethyldithiophosphate; DEP, diethylphosphate; DETP, dietylthiophosphate; DETDP, diethyldithiophosphate; NEO/m, neonicotinoid and their metabolites; ACE, acetamiprid; CLO, clothianidin; DIN, dinotefuran; IMI, imidacloprid; NIT, nitenpyram; THI, thiacloprid; THX, thiamethoxam; SUF, sulfoxaflor; FLN, flonicamid; N-dm-ACE, N-desmethyl-ACE; THI-AM, thiacloprid-amide; Of-IMI, imidacloprid-olefin; TFNA-AM, trifluoromethyl-nicotinamide; OH-IMI, hydroxy-IMI; N-dm-THX, N-desmethyl-THX; -, values under MDLs. Note: MDLs were 2.5 μg/L for DMP; 1.0 μg/L for Of-IMI; 0.2 μg/L for DMTP, DMDTP, DEP, DEDTP, and TZNG; 0.1 μg/L for DETP, FLN, N-dm-THX, OH-IMI, TFNA-AM, and fipronil; 0.05 μg/L for CLO, DIN, IMI, NIT, and N-dm-ACE; 0.02 μg/L for THX, fipronil sulphide, fipronil sulfone, and ethiprole; 0.01 μg/L for THIAM; and 0.005 μg/L for ACE, THI, and SUF.

**Table 2:** Predictors of urinary concentrations of DAP in reproductive-age farmworkers (N = 100).

Variables	DEP	DETP	IMI	N-dm-ACE
	β (95% CI)	β (95% CI)	β (95% CI)	β (95% CI)
Adjusted R^2^	0.400	0.256	0.131	0.110
(Intercept)	0.47 (0.16, 0.78)	0.27 (−0.19, 0.74)	−0.89 (−1.50, −0.28)	−0.33 (−0.75, 0.09)
Gender				
Female	Reference		Reference	
Male	−0.09 (−0.21, 0.03)	−0.08 (−0.26, 0.10)	−0.07 (−0.31, 0.16)	−0.20 (−0.37, −0.04)
Age	0.07 (0.0001, 0.14)	0.08 (−0.03, 0.18)	0.08 (−0.06, 0.22)	−0.04 (−0.13, 0.06)
BMI	−0.06 (−0.12, 0.01)	−0.06 (−0.16, 0.04)	0.03 (−0.10, 0.17)	0.03 (−0.06, 0.13)
Study site area				
Chom Thong District	Reference		Reference	
Fang District	0.32 (0.11, 0.53)	0.31 (−0.001, 0.62)	0.13 (−0.27, 0.54)	−0.13 (−0.41, 0.15)
Educational level				
No formal education	Reference		Reference	
Primary school	−0.05 (−0.23, 0.13)	−0.03 (−0.29, 0.24)	0.13 (−0.22, 0.48)	0.03 (−0.21, 0.28)
High school	−0.13 (−0.36, 0.10)	−0.18 (−0.53, 0.16)	0.12 (−0.33, 0.57)	−0.13 (−0.44, 0.18)
More than high school	0.04 (−0.20, 0.29)	0.001 (−0.36, 0.36)	0.16 (−0.32, 0.63)	0.35 (0.02, 0.68)
Family income	0.03 (−0.04, 0.11)	0.10 (−0.01, 0.21)	0.06 (−0.09, 0.20)	0.09 (−0.01, 0.19)
Occupation status				
Working their own farm	Reference		Reference	
Working on rented farm	−0.09 (−0.31, 0.12)	0.04 (−0.37, 0.28)	0.05 (−0.37, 0.47)	−0.03 (−0.32, 0.26)
Working in others’ farm	0.04 (−0.16, 0.24)	0.02 (−0.28, 0.31)	0.39 (0.0004, 0.77)	0.17 (−0.10, 0.43)
Residential pesticide use				
No	Reference		Reference	
Yes	0.13 (−0.02, 0.29)	0.20 (−0.04, 0.43)	0.08 (0.23, 0.38)	0.03 (−0.18, 0.24)
Home-grown vegetables consumption				
No	Reference		Reference	
Yes	0.10 (−0.04, 0.25)	0.22 (0.003, 0.44)	0.12 (−0.16, 0.41)	−0.08 (−0.28, 0.12)
Home-grown fruits consumption				
No	Reference		Reference	
Yes	−0.09 (−0.23, 0.04)	0.14 (−0.35, 0.06)	−0.002 (−0.27, 0.27)	0.14 (−0.05, 0.32)
Source of water consumption				
Tap water	Reference		Reference	
Stream water	0.07 (−0.17, 0.30)	0.15 (−0.21, 0.50)	0.004 (−0.46, 0.47)	0.09 (−0.23, 0.41)
Bottled water	−0.04 (−0.33, 0.24)	0.05 (−0.38, 0.47)	0.08 (−0.48, 0.63)	−0.01 (−0.39, 0.37)
Well water	0.44 (0.08, 0.80)	0.57 (0.03, 1.12)	0.98 (0.27, 1.69)	0.02 (−0.47, 0.51)
Filtered water	0.09 (−0.17, 0.34)	0.03 (−0.35, 0.41)	0.16 (−0.33, 0.66)	−0.16 (−0.50, 0.19)

Abbreviations: β, standardised partial regression coefficient; CI, confidence interval; R^2^, coefficient of determination; DEP, diethylphosphate; DETP, diethylthiophosphate; IMI, imidacloprid; N-dm-ACE, N-desmethyl-acetamiprid.

## Supplementary Materials

The following materials are available online at https://www.mdpi.com/1660-4601/17/21/7871/s1, Table S1: Urinary dialkylphosphate (DAP) concentrations in German External Quality Assessment Scheme (G-EQUAS) materials batch no. 64/2019; Table S2: Sociodemographic characteristics of farmworkers (N = 100), Table S3: Work and exposure characteristics of farmworkers (N = 100), Table S4: Work and exposure characteristics of farmworkers: agricultural activities (N = 100), Table S5: Work and exposure characteristics of farmworkers: personal protective equipment (PPE) used (N = 100).
