# Supplementary material for: Characteristics of Exposure of Reproductive-Age Farmworkers in Chiang Mai Province, Thailand, to Organophosphate and Neonicotinoid Insecticides: A Pilot Study
Source: Int J Environ Res Public Health. 2020 Oct 27;17(21):7871. doi: 10.3390/ijerph17217871 (PMC7663695; doi:10.3390/ijerph17217871)
Supplement: Supplementary file 1 [file ijerph-17-07871-s001.pdf]

Supplementary materials

Table S1. Urinary dialkylphosphate (DAP) concentrations in the German External Quality Assessment Scheme (G-EQUAS) materials batch no. 64/2019.

**Analyte**	**Level A**			**Level B**		
	**Reported value**	**Reference value**	**Tolerance range**	**Reported value**	**Reference value**	**Tolerance range**
DMP	18.34	18.12	12.51–23.73	58.73	71.79	55.20–88.38
DMTP	13.61	11.98	7.69–16.27	28.46	30.61	22.45–38.77
DMDTP	2.64	2.91	1.86–3.96	3.9	4.61	3.11–6.11
DEP	4.74	4.98	3.27–6.69	37.41	45.64	32.80–58.48
DETP	16.92	15.33	9.54–21.12	86.05	87.7	70.75–104.65
DEDTP	0.88	0.68	0.38–0.98	2.53	2.03	1.34–2.72

Abbreviation: DMP, dimethylphosphate; DMTP, dimetylthiophosphate; DMTDP, dimethyldithiophosphate; DEP, diethylphosphate; DETP, dietylthiophosphate; DETDP, diethyldithiophosphate. Note: Analyte concentrations reported as µg/L (or ng/mL).

Table S2. Sociodemographic characteristics of farmworkers (*N* = 100).

**Sociodemographic characteristics**	**Female**	**Male**	**Total**
	***N* (%)**	***N* (%)**	***N* (%)**
Age, yrs (mean ± SD)	29.1 ± 5.7	31.2 ± 5.2	30.1 ± 5.6
18–30 years	24 (48)	20 (40)	44 (44)
31–40 years	26 (52)	30 (60)	56 (56)
Body mass index (BMI) (mean ± SD)	24.6 ± 4.1	24.3 ± 3.7	24.4±3.9
≤18.49 (underweight)	5 (10)	1 (2)	6 (6)
18.50–22.99 (normal weight)	14 (28)	20 (40)	34 (34)
23.00–24.99 (overweight)	9 (18)	10 (20)	19 (19)
25.00–29.99 (obese class I)	16 (32)	15 (30)	31 (31)
≥30.00 (obese class II)	6 (12)	4 (8)	10 (10)
Family income (USD/month) (mean ± SD)	320 ± 261	320 ± 261	320 ± 260
(THB/month) (mean ± SD)	(10012 ± 8139)	(10012 ± 8139)	(10012 ± 8098)
Ethnicity			
Northern Thai	20 (40)	20 (40)	40 (40)
Other	30 (60)	30 (60)	60 (60)
Education			
No formal education	17 (34)	12 (24)	29 (29)
Primary school	11 (22)	15 (30)	26 (26)
High school	16 (32)	15 (30)	31 (31)
More than high school	6 (12)	8 (16)	14 (14)
Marital status			
Married	13 (26)	13 (26)	26 (26)
Living as married	37 (74)	37 (74)	74 (74)
Parity			
0	2 (4)		
1	19 (38)		
>1	29 (58)		

Abbreviations: SD, standard deviation; USD, United States Dollar; THB, Thai Baht. Results reported as mean ± SD or *N* (%).

Table S3. Work and exposure characteristics of farmworkers (*N* = 100).

Work and exposure characteristics	Female	Male	Total
	N (%)	*N* (%)	*N* (%)
Total years as farmworker (mean ± SD)	11.5 ± 7.4	13.2 ± 6.7	12.3 ± 7.1
≤5 year	9 (18)	6 (12)	15 (15)
6–10 years	41 (82)	43 (86)	80 (80)
11–20 years	0 (0)	1 (2)	0 (0)
>20 years	0 (0)	0 (0)	5 (5)
Hours/day working in the field (mean ± SD)	8.3 ± 2.8	8.5 ± 2.0	8.4 ± 2.4
Occupational status			
Working own farm or family farm	36 (72)	37 (74)	73 (73)
Working on rented farm	5 (10)	5 (10)	10 (10)
Working on another person's farm	9 (18)	8 (16)	17 (17)
Residential pesticide used			
Yes	36 (72)	35 (70)	71 (71)
No	14 (28)	15 (30)	29 (29)
Consumption of home-grown vegetables			
Yes	36 (72)	33 (66)	69 (69)
No	14 (28)	17 (34)	31 (31)
Consumption of home-grown fruits			
Yes	23 (46)	24 (48)	47 (47)
No	27 (54)	26 (52)	53 (53)
Main source of water for consumption			
Tap water	5 (10)	5 (10)	10 (10)
Stream water	26 (52)	26 (52)	52 (52)
Bottled water	9 (18)	8 (16)	17 (17)
Water well	3 (6)	3 (6)	6 (6)
Filtered water	7 (14)	8 (16)	15 (15)

Abbreviations: SD, standard deviation. Results reported as mean ± SD or *N* (%).

Table S4. Work and exposure characteristics of farmworkers: agricultural activities (*N* = 100).

Work and exposure characteristics	Female	Male	Total
	N (%)	*N* (%)	*N* (%)
Planting			
Yes	43 (86)	45 (90)	88 (88)
No	7 (14)	5 (10)	12 (12)
Weeding			
Yes	46 (92)	45 (90)	81 (81)
No	4 (8)	5 (10)	9 (9)
Harvesting			
Yes	47 (94)	48 (96)	95 (95)
No	3 (6)	2 (4)	5 (5)
Applying fertilizer			
Yes	30 (60)	46 (92)	81 (81)
No	20 (40)	4 (8)	9 (9)
Watering			
Yes	38 (76)	47 (94)	94 (94)
No	12 (24)	3 (6)	6 (6)
Farm tasks related to pesticide use			
Preparing/mixing pesticides			
Yes	30 (60)	37 (74)	67 (67)

No	20 (40)	13 (26)	33 (33)
Handling containers of pesticides			
Yes	31 (62)	39 (78)	70 (70)
No	19 (38)	11 (22)	30 (30)
Spraying pesticides			
Yes	38 (76)	45 (90)	83 (83)
No	12 (24)	5 (10)	17 (17)

Results reported as N (%).

Table S5. Work and exposure characteristics of farmworkers: personal protective equipment (PPE) used (*N* = 100).

Work and exposure characteristics	Female	Male	Total
	***N* (%)**	***N* (%)**	***N* (%)**
Wearing mask			
Yes	38 (76)	40 (80)	78 (78)
No	12 (24)	20 (20)	22 (22)
Wearing hat or scarf as head protection			
Yes	28 (56)	25 (50)	53 (53)
No	22 (44)	25 (50)	27 (27)
Wearing glasses			
Yes	4 (8)	5 (10)	9 (9)
No	46 (82)	45 (90)	91 (91)
Wearing long-sleeved shirts/pants			
Yes	41 (82)	45 (90)	86 (86)
No	9 (18)	5 (10)	14 (14)
Wearing gloves			
Yes	34 (68)	32 (64)	66 (66)
No	16 (32)	18 (36)	34 (34)
Wearing boots			
Yes	38 (76)	45 (90)	83 (83)
No	12 (24)	5 (10)	17 (17)

Results reported as *N* (%).
